# Community knowledge, perceptions and attitudes regarding leprosy in rural Cameroon: The case of Ekondotiti and Mbonge health districts in the South-west Region

**DOI:** 10.1371/journal.pntd.0006233

**Published:** 2018-02-12

**Authors:** Earnest Njih Tabah, Dickson Shey Nsagha, Anne-Cécile Zoung-Kanyi Bissek, Theophilus Ngeh Njamnshi, Irine Ngani-Nformi Njih, Gerd Pluschke, Alfred Kongnyu Njamnshi

**Affiliations:** 1 National Yaws, Leishmaniasis, Leprosy and Buruli ulcer Control Programme, Department of disease, epidemics and pandemics control, Ministry of Public Health, Yaounde, Cameroon; 2 Department of Internal Medicine and Specialties, Faculty of Medicine and Biomedical Sciences, The University of Yaounde 1, Yaounde, Cameroon; 3 Department of Medical Parasitology and Infection Biology, Swiss Tropical and Public Health Institute, Basel, Switzerland; 4 Faculty of Science, University of Basel, Basel, Switzerland; 5 Department of Public Health and Hygiene, Faculty of Health Sciences, University of Buea, Buea, Cameroon; 6 Brain Research Africa Initiative (BRAIN), Yaounde, Cameroon; 7 Division of Operational Research in Health, Ministry of Public Health, Yaounde, Cameroon; 8 Department of Medical Laboratory Sciences, School of Health and Medical Sciences, Catholic University of Cameroon, Bamenda; 9 Department of International Development, Lead Higher Institute, Yaounde, Cameroon; 10 Department of Neurology, Central Hospital Yaounde, Yaounde, Cameroon; University of California San Diego School of Medicine, UNITED STATES

## Abstract

**Background:**

Although leprosy is one of the oldest diseases known to humanity, it remains largely misunderstood. Misconceptions about leprosy lead to stigma towards people with the disease. This study aimed at exploring the knowledge, perceptions and attitudes regarding leprosy in rural Cameroon.

**Methods:**

We carried out a cross-sectional community survey of 233 respondents aged 15–75 years, free from leprosy, and living in two rural health districts of the South-west Region of Cameroon. A questionnaire designed to evaluate knowledge, perceptions and attitudes about leprosy was used. Binary logistic regression was used to determine independent predictors of negative attitudes.

**Results:**

About 82% of respondents had heard about, and 64.4% knew someone with leprosy. Information on leprosy was mainly from community volunteers (40.6%), friends (38.0%), and the media (24%). Only 19.7% of respondents knew the cause of leprosy, and a considerable proportion linked it to a spell (25.3%), unclean blood (15.5%) and heredity (14.6%). About 72% knew that leprosy is curable and 86.3% would advise medical treatment. Attitudes towards leprosy patients were generally negative. Only 42% would shake hands, 32.6% would share the same plate, and 28.3% and 27% respectively, would allow their child to play or marry a person with leprosy. Furthermore, only 33.9% approved of participation of leprosy patients, and 42.9% of their employment. Independent predictors of negative attitudes were: the belief that leprosy is a curse; is caused by a germ; and having seen a leprosy patient. The negative attitudes were dampened by: the beliefs that leprosy is a punishment, is hereditary and is due to poor personal hygiene.

**Conclusion:**

An awareness intervention using community volunteers and the media, with information on the cause of leprosy, its clinical manifestations and curability, and sensitization messages correcting the misconceptions and beliefs regarding leprosy, could improve the community knowledge and attitudes towards leprosy. This would ultimately contribute to the reduction of leprosy burden in the community.

## Author summary

Leprosy is one of the oldest diseases known to humanity but remains largely misunderstood. This misunderstanding leads to stigma towards people with leprosy (PWL). We explored knowledge, perceptions and attitudes regarding leprosy among 233 community members in the South-west of Cameroon. Our respondents were very familiar with leprosy. Their information on leprosy was mainly from community volunteers, friends or from the media. Despite high familiarity, very few knew the cause of leprosy. A good proportion attributed it to curses, unclean blood, or heredity. However, most of them agreed that leprosy was curable and would advise medical treatment. Attitudes of community members towards PWL were generally negative. Very few of them would shake hands with, eat from the same plate, or allow their child to play with or marry a PWL. The main reasons for these negative attitudes were the beliefs that leprosy is a curse; is caused by a germ; and having seen a leprosy patient. An awareness campaign using community volunteers and the media, with information on the cause of leprosy, its clinical manifestations and curability could improve community knowledge and attitudes towards leprosy. This would ultimately contribute to the reduction of leprosy burden in the community.

## Introduction

Leprosy is one of the oldest diseases known to humanity, and can be traced as far back as 100 000 years [1]. It is an infectious disease caused by *Mycobacterium leprae*. It affects peripheral nerves, the skin and the mucosa of the upper respiratory pathways [2]. Although the exact mode of transmission is not clear, it is believed to occur through nasal droplets or prolonged skin contact with an untreated patient [3; 4].

For a long time, humans were believed to be the only reservoir of *Mycobacterium leprae*. However, since 2005 the 9-banded armadillos in southcentral [5] and south-eastern [6] United States of America were confirmed to harbour the bacilli and to transmit it amongst themselves [6]. Another rodent, the red squirrels in the British Isles has also been shown to harbour the bacilli [7]. These new findings have implications for zoonotic transmission of leprosy [5; 6] as well as for the eradication of this scourge [8].

Untreated leprosy patients or those with late diagnosis usually develop irreversible and progressive disabilities and disfiguring complications. Physical deformities in addition to socio-cultural misconceptions about leprosy have led to intense social stigma and discrimination of people with leprosy (PWL) throughout history [9; 10; 11]. Social stigma related to leprosy is typically anticipated, felt or experienced by the victim [9] and is generally characterised by social exclusion, rejection, blame, and participation restriction among others [12; 13; 11]. Social stigma has been blamed for delay in seeking treatment by leprosy patients, who because of anticipated stigma, would rather prefer to conceal their condition [14; 15]. This has been an obstacle to early detection, prompt treatment and cure of leprosy patients.

Despite the advances in treatment [16; 17] and political commitment at the global level [18] with attendant reduction in leprosy burden worldwide [19], further reduction of leprosy burden meets with enormous challenges. These challenges are three-prong, including further reduction in the number of new cases, the registered prevalence, and the social stigma and exclusion through prevention and management of disabilities [20]. The full involvement of endemic communities as well as persons affected by leprosy is primordial in these efforts of leprosy burden reduction [20].

In Cameroon, leprosy elimination was achieved at the national level since 2000.The current prevalence and detection rates are below 0.20/10 000 and 1.46/100 000 population respectively [21]. By the end of 2014, the proportion of MB leprosy among new cases was 87%, the proportion of child cases was 18%, and the female proportion was 43%. The grade-2-disability proportion was 7% and the rate was 0.10/100 000 population [21]. In addition, ten health districts (HD) remained highly endemic for leprosy by the end of 2014 [21].

In order to assist the national leprosy control programme (NLCP) to improve the strategies for further reduction of the leprosy burden, we carried out a community-based study to assess knowledge, perceptions and attitudes regarding leprosy in the Ekondotiti and Mbonge HDs in the South-west Region of Cameroon.

## Methods

### Study design

We carried out a community-based cross-sectional descriptive and analytical study of knowledge, perceptions and attitudes regarding leprosy in rural Cameroon. The study was done within the framework of a screening campaign for leprosy and other skin diseases in Ekondotiti and Mbonge HDs of the South-west Region of Cameroon, organized by the NLCP (results presented elsewhere).

### Survey setting

**Fig 1:**
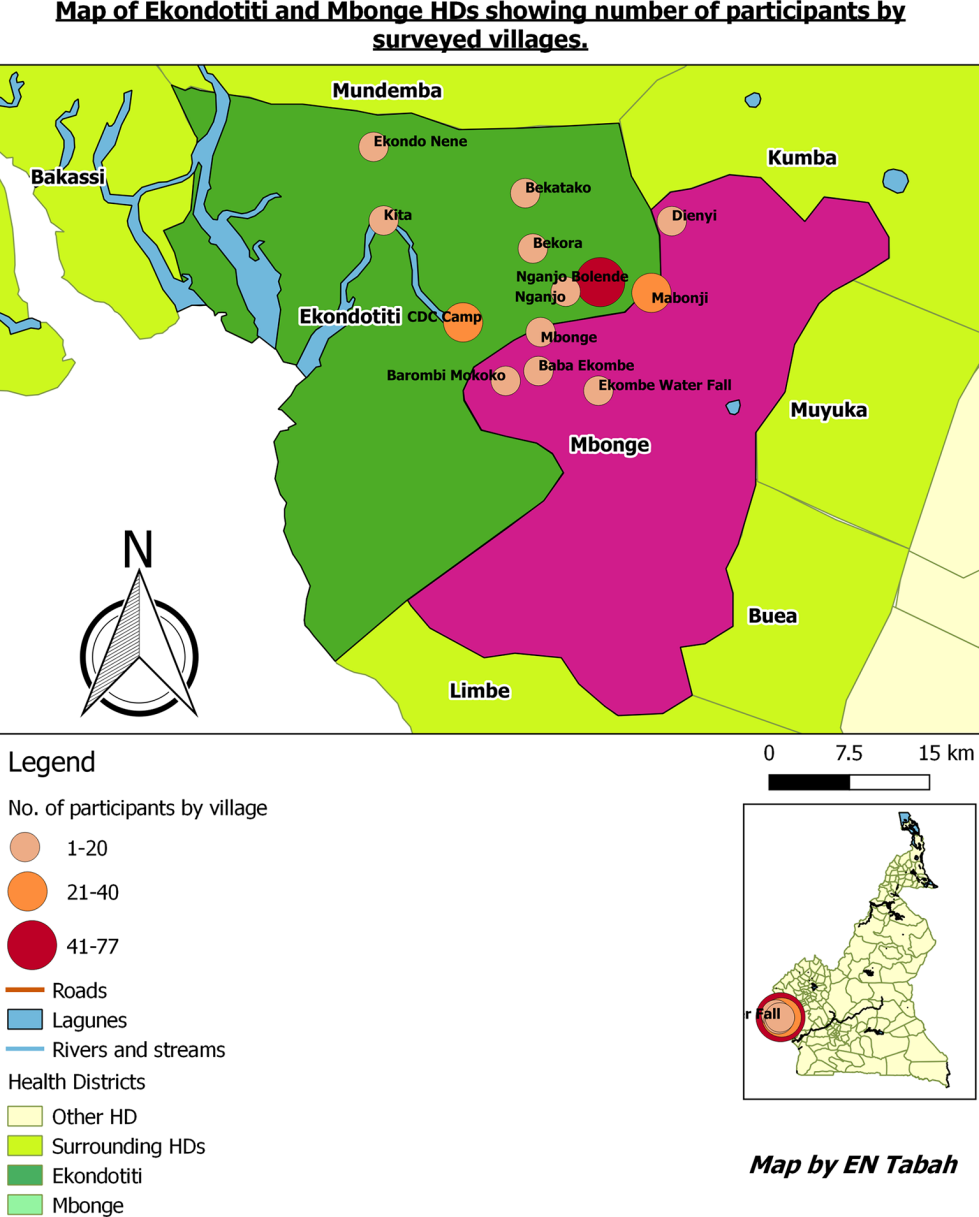
Map of Ekondotiti and Mbonge health districts showing number of participants by village surveyed (drawn using QGIS version 2.18.9 Las Palmas [22]).

This community-based survey was carried out in April and May 2015 in two neighbouring rural HDs of Ekondotiti and Mbonge of the South-west Region of Cameroon (Fig 1). These districts were among those with the highest leprosy-burden in the country between 2010 and 2014 [21]. Ekondotiti and Mbonge HDs comprise 78 and 65 villages respectively. Six villages from Ekondotiti and seven from Mbonge respectively, were selected for the survey, based on leprosy case-notification from 2010–2014.

**Fig 2:**
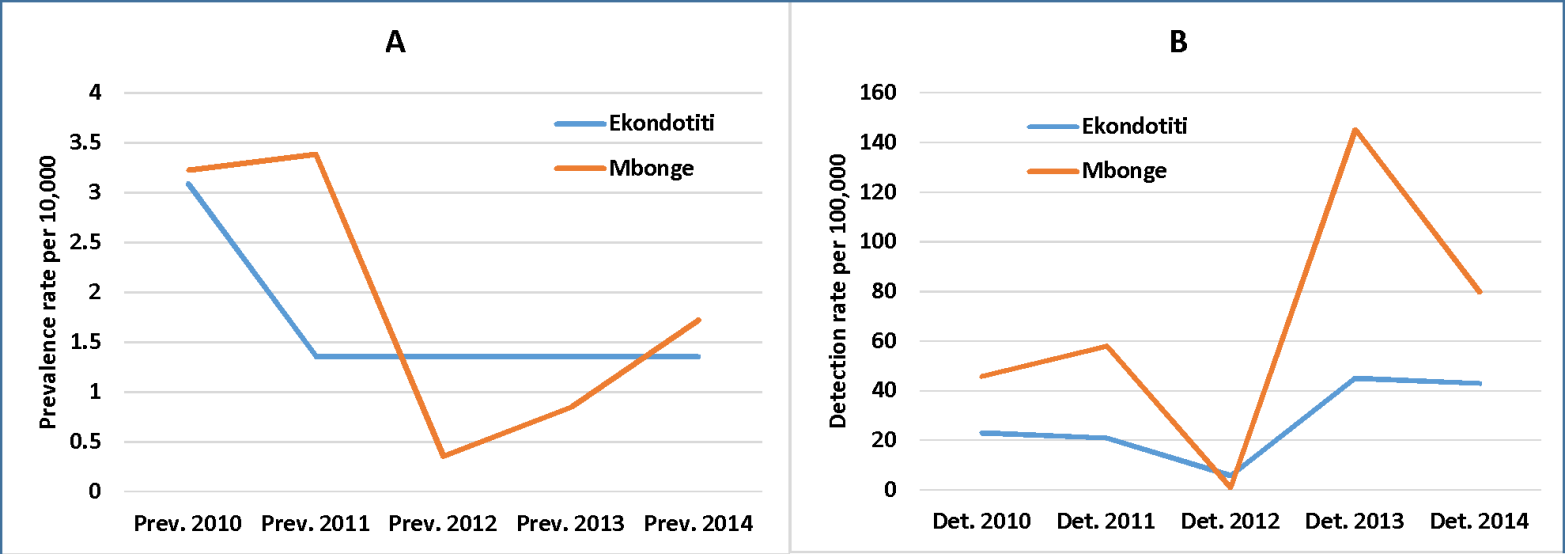
Panel A shows the trend in the leprosy prevalence rate per 10,000 populations from 2010 to 2014, while panel B shows the leprosy detection rate per 100,000 populations over the same period in Mbonge and Ekondotiti health districts. The trend in leprosy prevalence rate was constantly above 1 per 10000 populations in Ekondotiti. For Mbonge, it fluctuated from 3.23 in 2010 down to 0.36 in 2012 and back to 1.73 per 10000 populations in 2014. Over the same period, the leprosy detection rate was stable at about 21 per 100,000 populations in Ekondotiti from 2010–2011, then dropped to 6 in 2012 before rising again to 43.1 per 100,000 in 2014. In Mbonge, the detection witnessed fluctuations from about 50 per 100,000 populations between 2010 and 2011, down to 1.2 in 2012, then rose sharply to 145.5 in 2013 before dropping again to 80 in 2014.

The 2010–2014 trend in leprosy prevalence rate was constantly above 1 per 10000 populations in Ekondotiti. For Mbonge, it fluctuated from 3.23 in 2010 down to 0.36 in 2012 and back to 1.73 per 10000 population in 2014 (Fig 2A). Over the same period, the leprosy detection rate was stable at about 21 per 100,000 population in Ekondotiti from 2010–2011, then dropped to 6 in 2012 before rising again to 43.1 per 100,000 in 2014. In Mbonge, the detection rate was higher than in Ekondotiti but witnessed fluctuations from about 50 per 100,000 populations between 2010 and 2011, down to 1.2 in 2012, then rose sharply to 145.5 in 2013 before dropping again to 80 in 2014 (Fig 2B).

Three-quarters of the inhabitants of Ekondotiti and Mbonge HDs were of the Oroko tribe, sub-divided into ten clans [23], with each clan speaking their own dialect [24]. Despite the predominance of Oroko people, the two HDs are quite cosmopolitan, with inhabitants from diverse ethnic origins of Cameroon. With this mix, the use of Pidgin English language has been highly developed and is widespread in the area [25]. The two HDs fall within the cocoa production basin of the South-west Region and majority of the inhabitants are farmers, involved mainly in cocoa farming.

### Participants, sampling and data collection

#### Participants

Participants included in the study were individuals of both sexes aged 15 years and older. Persons below 15 years of age, leprosy patients, health care personnel and those who did not give their consent were excluded from the study.

#### Sampling

The survey team visited 13 villages selected for the survey according to a pre-established schedule. In each village, villagers were invited to gather at a central place for screening of leprosy and other skin conditions. We used a systematic random sampling whereby every fifth person screened, who fulfilled the inclusion criteria and who gave his/her consent to participate in the survey was selected for interview.

#### Data collection

Data was collected using a closed ended questionnaire in English, designed to collect demographic variables and to evaluate knowledge, perceptions and attitudes regarding leprosy, adapted from the one used for KAP-epilepsy studies in Cameroon [26; 27; 28]. Four data collectors fluent in both English and the Pidgin-English languages were trained on the administration of the questionnaire by the lead author. The training included among other things, full understanding of, and appropriate translation of the questionnaire into Pidgin-English and back-translation into the English language by the data collectors. The questionnaire was field-tested in two villages which were not included for the survey. After field testing, questions 5 and 7 were modified for better comprehension. The data collectors then moved along with the leprosy screening team and conducted face-to-face interviews with the participants.

## Operational definitions and outcome variables

### Operational definitions

#### High knowledge of leprosy

Participants who answered “Yes” to ≥ 50% of knowledge questions were considered as having high knowledge.

#### Positive attitudes

Participants who answered “Yes” to ≥ 50% of the attitude questions were considered having positive attitudes toward PWL.

#### Erroneous perceptions

Participants who indicated any of the following (curse, bad blood, heredity, divine punishment, marrying from a family with history of leprosy) as being the cause of leprosy and/or who believed that leprosy is not curable was considered as having erroneous perceptions regarding leprosy.

### Outcome variables

The questionnaire designed for the survey included fifteen questions: 7 to assess knowledge and perceptions and 8 to assess attitudes regarding leprosy (Table 1).

**Table 1:** List of study outcome variables.

	Variable number	Variable (Question)
Knowledge and perceptions regarding leprosy	*Q1	Have you heard about leprosy?
	Q2	Have you ever seen someone with leprosy?
	Q3	Do you know someone with leprosy?
	Q4	Do you have a relative who has or had leprosy?
	Q5	What is the cause of leprosy according to you? (Yes = Germ/microbe, poor hygiene, living in close contact with a leprosy patient, No: any other cited cause)
	Q6	Do you think leprosy is curable?
	Q7	Where would you advise your relative or friend to seek treatment if he/she had leprosy? (Yes = health facility, medical doctor, nurse; No = Roadside medicine, No treatment, Traditional/spiritual healer)
Attitudes regarding leprosy	Q8	Would you shake hands with someone with leprosy?
	Q9	Would you eat from the same plate with someone with leprosy?
	Q10	Would you feel ashamed if you had leprosy?
	Q11	Would you reveal your status to someone if you had leprosy?
	Q12	Would you allow your child to play with another child who has/had leprosy?
	Q13	Would you accept your child to marry from a family with a history of leprosy?
	Q14	Do you think people who have/had leprosy should be allowed to participate in activities like anyone else?
	Q15	Do you think people who have/had leprosy should be given employment like anyone else?

*Q = Question

#### Sample size

Based on an assumed proportion for negative attitudes towards lepers of 21.6% demonstrated in the northwest of Cameroon [13], and for a 95% confidence interval, and an acceptable error of 0.05, a sample size of 261 was determined for our study.

### Ethics statement

Ethical approval was obtained from the National Ethics Committee for Research in Human Health, Yaounde, Cameroon (N° 172/CNE/SE/2011). Participation in the study was voluntary and each participant gave an informed consent. All data were anonymized and confidentiality was strictly respected in the data handling and analysis.

### Data management and statistical methods

Data management consisted of checking whether questionnaires were filled completely and correctly using appropriate codes. This was done daily until all the data was collected. The data was stored in a safe place until analysed.

Data was entered on Microsoft Excel spread sheets and exported to SPSS for Windows version 20 statistical software for analysis. Proportions were calculated and the Chi-square test was used to examine associations between responses and variables. The level of significance was set at p <0.05. After performing orienting univariate analyses, we carried out binary logistic regression analysis to determine predictors of negative attitudes.

## Results

### Characteristics of participants

Two hundred and sixty-one (261) individuals were contacted and 233 accepted to participate in the survey, giving a response rate of 89.3%. Their ages ranged from 15 to 75 years with a mean age of 33 ± 12 years. They were 118 (50.6%) males. Seventy-two percent were protestant Christians. The majority (65.7%) were from the Oroko tribe, while 34.3% of them originated from 21 other Cameroonian tribes. Most (59.7%) of the participants had only the primary level of education, 56.7% were married and 59.2% of them were farmers.

### Knowledge, beliefs and perceptions regarding leprosy

The details of familiarity with and knowledge of leprosy are shown in Table 2. Generally, our respondents were very familiar with leprosy, as 82.4% had heard about it and 64.4% had seen someone with the condition. About 75% of them declared that leprosy was curable however; only 19.7% knew the cause of the disease.

**Table 2:** Relationship between knowledge, beliefs and perceptions regarding leprosy and demographic variables.

	N° of respondents	Q1		Q2		Q3		Q4		Q5		Q6	
Total	233	82.4		64.4		46.4		11.2		19.7		75.1	
***Age group***													
10–19yrs	17	70.6		**29.4**		**29.4**		5.9		17.6		58.8	
20–29yrs	91	79.1		51.6		36.3		6.6		17.6		74.7	
30–39yrs	60	90.0	P = 0.300	76.7	**P < 0.001**	56.7	**P = 0.033**	**23.3**	**P = 0.005**	28.3	P = 0.368	81.7	P = 0.484
40–49yrs	39	84.6		76.9		53.8		2.6		17.9		74.4	
50+yrs	26	80.8		84.6		57.7		15.4		11.5		73.1	
***Sex***													
Female	115	82.6	P = 0.935	**55.7**	**P = 0.006**	**38.3**	**P = 0.014**	7.8	P = 0.111	19.1	P = 0.817	76.5	P = 0.708
Male	118	82.2		72.9		54.2		14.4		20.3		73.7	
***Level of Education***													
None	13	61.5		**46.2**		38.5		7.7		0.0		53.8	
Primary	139	83.5	P = 0.246	61.2	**P = 0.041**	44.6	P = 0.738	13.7	P = 0.466	19.4	P = 0.255	77.7	**P = 0.047**
Secondary	57	84.2		66.7		49.1		8.8		24.6		70.2	
High school /University	24	83.3		87.5		54.2		4.2		20.8		**83.3**	
***Occupation***													
Business	36	80.6		61.1		44.4		8.3		22.2		**91.7**	
Farmer	138	81.9		63.8		45.7		11.6		21.0		70.3	
Pupil/Student	8	62.5	P = 0.458	75.0	P = 0.175	50.0	P = 0.739	12.5	P = 0.970	12.5	P = 0.891	75.0	**P = 0.043**
Salaried worker	30	90.0		80.0		56.7		13.3		16.7		86.7	
Unemployed	21	85.7		47.6		38.1		9.5		14.3		61.9	
***Marital status***													
Widowed/divorced	15	66.7		66.7		60.0		6.7		6.7		66.7	
Married	132	85.6	P = 0.151	71.2	**P = 0.028**	51.5	**P = 0.045**	15.2	P = 0.086	22.7	P = 0.266	79.5	P = 0.500
Single	86	80.2		**53.5**		**36.0**		5.8		17.4		69.8	
***Religion***													
Animist/Pagan	19	84.2		68.4		47.4		10.5		5.3		68.4	
Catholic Christian	44	77.3	P = 0.465	63.6	P = 0.955	40.9	P = 0.885	11.4	P = 0.378	27.3	P = 0.205	84.1	P = 0.740
Muslim	2	50.0		50.0		50.0		50.0		0.0		100.0	
Protestant Christian	168	83.9		64.3		47.6		10.7		19.6		73.2	

Figures under the question columns represent percentages of participants with a "Yes" response to the question

The knowledge of leprosy and its cause were not influenced by demographic variables. Regarding familiarity with leprosy, respondents below 20 years of age (p<0.001), females (p = 0.006), those with no level of formal education (p = 0.041), and singles (p = 0.028) were least likely to have seen someone with leprosy. Those below 20 years of age (p = 0.033), females (p = 0.014), and singles (p = 0.045) were least likely to know someone with the condition (Table 2). We found the highest proportion of respondents in the group aged 30–39 years (p = 0.005) who reported having a relative with leprosy. The unemployed (p = 0.043) and those with no level of formal education (p = 0.047) were the least likely to know that leprosy is curable (Table 2).

**Fig 3:**
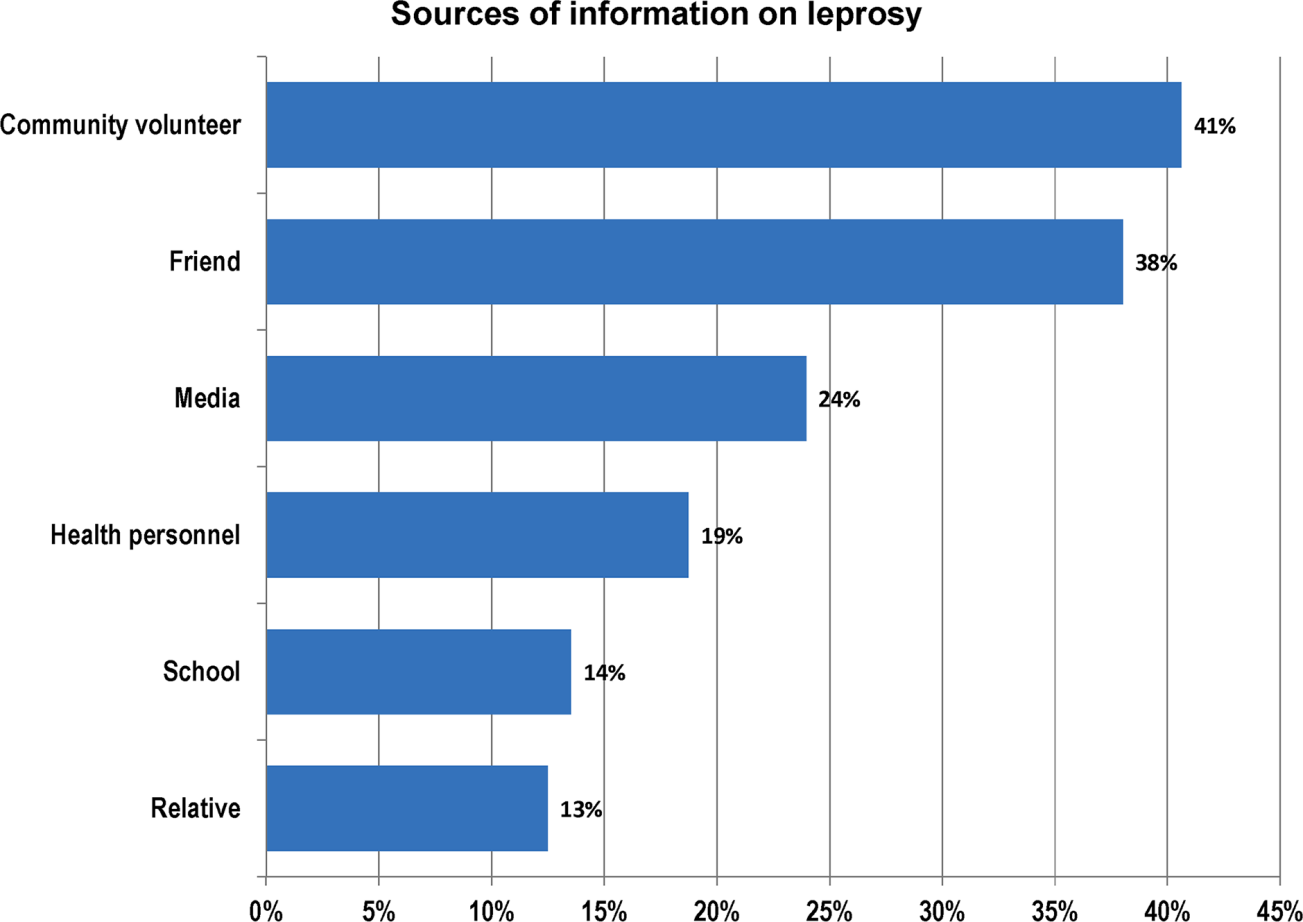
Sources of information on leprosy. The major sources of information on leprosy to our participants were from community volunteers (41%), friends (38%), the media (24%), and health personnel (19%).

For the 192 (82.4%) respondents who declared having heard about leprosy, their main sources of information on leprosy were from community volunteers (40.6%), friends (38.0%) and the media (24.0%) (Fig 3).

The beliefs and perceptions held about leprosy in the Mbonge and Ekondotiti HDs are portrayed in the nature of causes cited by the respondents (Table 3). Although 29%, 27% and 10.3% of them respectively rightly linked leprosy to germs, poor personal hygiene, and living in close contact with an untreated leprosy patient, a considerable proportion cited erroneous causes. A considerable proportion of them believed that leprosy is a spell (25.3%), is caused by unclean blood (15.5%), is hereditary (14.6%), or results from marrying from a family that has/had leprosy (11.2%). A much lesser proportion of the respondents believed that leprosy is punishment for sins, is caused by natural forces, or results from eating some food types or from malnutrition.

**Table 3:** Causes of leprosy as cited by the respondents.

Cited causes of leprosy	N° of respondents	Proportion of ‘Yes’ responses
Poor personal hygiene	233	28.8%
Germs or microbes	233	27.0%
Curse or spell	233	25.3%
Bad or unclean blood	233	15.5%
Heredity	233	14.6%
Marrying from a family that has/ had a leprosy patient	233	11.2%
Living in close contact with an untreated leprosy patient	233	10.3%
Spontaneous occurrence	233	8.6%
Divine punishment for sin	233	8.2%
Some natural forces	233	6.4%
Malnutrition	233	6.4%
Some types of food	233	4.3%

### Problems faced by people with leprosy or their families

**Fig 4:**
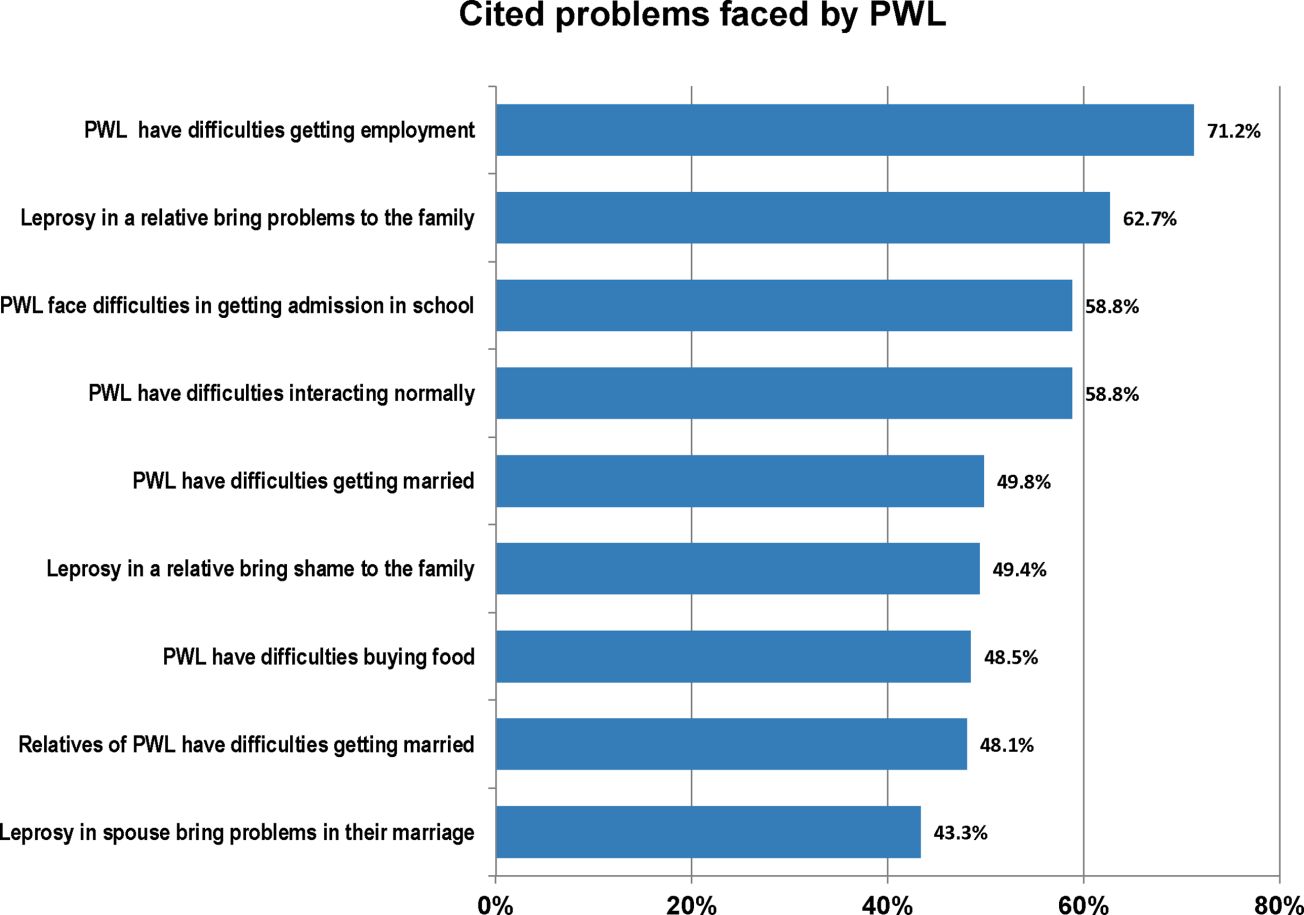
Cited problems faced by people with leprosy. The participants of our study admitted that PWL face a variety of problems in the society, ranging from difficulties getting employment, admission in school, or getting married themselves; to bringing shame in the family and causing other problems to family members.

Between 43% and 71% of our respondents admitted that PWL and their families face a variety of problems, ranging from difficulties getting employment, admission in school, or getting married themselves; to bringing shame in the family and causing other problems to family members (Fig 4).

Table 4 shows details of attitudes regarding leprosy among our respondents. A high proportion (86.3%) of them would advise a relative or friend with leprosy to consult a health professional, and 58.8% would be willing to tell someone if they had leprosy. Most of our respondents portrayed very negative attitudes with respect to leprosy, as only 42% would shake hands, and 32.6% would eat from the same plate with a leprosy patient. Only 28.3% and 27% would allow their child play with another child who had leprosy, or marry from a family with a history of leprosy, respectively. Only 33.9% of our respondent approved of leprosy patients participating in activities like anyone else, and 42.9% agree that they should be employed normally. Attitudes generally were not influenced by demographic variables, except for pupils/students, who were the least likely to reveal their leprosy status to anyone (p = 0.019).

**Table 4:** Relationship between attitudes regarding leprosy and demographic variables.

	No. of respondents	Q7		Q8		Q9		Q10		Q11		Q12		Q13		Q14		Q15	
Total	233	86.3		42.5		32.6		39.1		58.8		28.3		27.0		33.9		42.9	
***Age group***																			
10–19yrs	17	82.4		47.1		35.3		17.6		35.3		35.3		23.5		23.5		41.2	
20–29yrs	91	84.6		33.0		22.0		46.2		60.4		24.2		25.3		31.9		42.9	
30–39yrs	60	91.7	P = 0.627	53.3	P = 0.074	40.0	P = 0.203	36.7	P = 0.203	66.7	P = 0.369	31.7	P = 0.905	25.0	P = 0.885	33.3	P = 0.691	35.0	P = 0.521
40–49yrs	39	82.1		53.8		35.9		43.6		59.0		30.8		35.9		46.2		59.0	
50+yrs	26	88.5		30.8		46.2		26.9		50.0		26.9		26.9		30.8		38.5	
***Sex***																			
Female	115	90.4	P = 0.068	45.2	P = 0.222	33.9	P = 0.081	33.9	P = 0.052	60.9	P = 0.081	27.8	P = 0.077	27.0	P = 0.081	35.7	P = 0.079	45.2	P = 0.611
Male	118	82.2		39.8		31.4		44.1		56.8		28.8		27.1		32.2		40.7	
***Level of Education***																			
None	13	69.2	P = 0.150	53.8	P = 0.057	53.8	P = 0.174	46.2	P = 0.828	46.2	P = 0.512	38.5	P = 0.486	38.5	P = 0.408	30.8	P = 0.243	38.5	P = 0.235
Primary	139	87.1		38.8		33.8		38.1		59.7		30.2		25.9		32.4		41.0	
Secondary	57	84.2		36.8		22.8		38.6		54.4		24.6		22.8		29.8		38.6	
High school /University	24	95.8		70.8		37.5		41.7		70.8		20.8		37.5		54.2		66.7	
***Occupation***																			
Business	36	97.2		47.2		38.9		38.9		63.9		30.6		38.9		47.2		47.2	
Farmer	138	82.6	P = 0.054	38.4		30.4		42.8		52.2		27.5		24.6		31.2		40.6	
Pupil/Student	8	75.0		25.0	P = 0.502	0.0	P = 0.388	12.5	P = 0.747	**25.0**	**P = 0.019**	0.0	P = 0.568	12.5	P = 0.562	0.0	P = 0.064	12.5	P = 0.568
Salaried worker	30	96.7		60.0		43.3		30.0		76.7		33.3		33.3		50.0		53.3	
Unemployed	21	81.0		42.9		33.3		38.1		81.0		33.3		19.0		19.0		47.6	
***Marital status***																			
Widowed/divorced	15	80.0		26.7		13.3		40.0		46.7		20.0		20.0		20.0		26.7	
Married	132	87.9	P = 0.630	43.9	P = 0.525	35.6	P = 0.199	36.4	P = 0.532	62.1	P = 0.343	28.8	P = 0.429	25.8	P = 0.353	37.9	P = 0.234	43.2	P = 0.482
Single	86	84.9		43.0		31.4		43.0		55.8		29.1		30.2		30.2		45.3	
***Religion***																			
Animist/Pagan	19	73.7		47.4		52.6		36.8		52.6		36.8		36.8		47.4		52.6	
Catholic Christian	44	86.4	P = 0.380	34.1	P = 0.547	20.5	P = 0.094	47.7	P = 0.872	59.1	P = 0.888	18.2	P = 0.682	27.3	P = 0.319	31.8	P = 0.892	36.4	P = 0.906
Muslim	2	100.0		100.0		100.0		50.0		100.0		50.0		100.0		50.0		50.0	
Protestant Christian	168	87.5		43.5		32.7		36.9		58.9		29.8		25.0		32.7		43.5	

Figures under the question columns represent percentages of participants with a "Yes" response to the question

The analysis of the effect of knowledge, beliefs and perceptions regarding leprosy of our respondents on their attitudes toward PWL is detailed in Table 5. The acceptance to refer a relative or friend with leprosy to a health facility was greater in respondents who knew or who had seen someone with leprosy (p = 0.026), and who understood that leprosy is caused by a germ (p = 0.014) and that it is curable (p<0.001). Only those who understood leprosy is curable declared they would shake hands with patients (p = 0.002). Those who had heard about leprosy (p = 0.041), and who understood that leprosy is curable (p = 0.002) were more likely to eat from the same plate with a patient, but those who thought leprosy was due to poor personal hygiene were least likely to do so (p = 0.042). Respondents who knew leprosy is curable were more likely to feel ashamed (p<0.001). Those who had heard about leprosy (p = 0.039) and who knew leprosy is curable were more likely to conceal their status (p<0.001) if they had leprosy, but those who believed leprosy is a punishment for sins (p = 0.005) or is caused by living in close contact with a patient (p = 0.027) were least likely to conceal their status if they were affected. Those who had heard about leprosy (0.039) and who understood it is curable (p = 0.014), or believed it was a punishment for sins (p = 0.011), were least likely to allow their children play with one who had leprosy. Respondents who had heard about leprosy (p = 0.026) were least likely to allow their children marry from a family with a history of leprosy, meanwhile those who knew leprosy is curable (p = 0.016) were readier to let their children marry from such a family. Those who had heard about leprosy (p = 0.034), who believed it was caused by living in close contact with an untreated patient (p = 0.018) or due to poor personal hygiene (0.022) were least likely to accept that leprosy patients participate in activities like anyone else. However, those who knew leprosy is curable (p = 0.005) had no problem with patients participating normally in activities. Concerning employment of PWL, those who had heard about the condition (p = 0.004), or who knew it was curable (p = 0.002) were more likely to offer them employment, but those who believed leprosy was hereditary (p = 0.033) or due to poor personal hygiene (p = 0.007) would not do so.

**Table 5:** Relationship between knowledge, beliefs and perceptions regarding leprosy and attitudes towards PWL.

		Number of respondents	Q7		Q8		Q9		Q10		Q11		Q12		Q13		Q14		Q15	
	Total	233	86.3		42.5		32.6		39.1		58.8		28.3		27.0		33.9		42.9	
Have you heard about leprosy?	No	41	78.0	P = 0.092	36.6	P = 0.092	29.3	**P = 0.041**	**48.8**	**P = 0.026**	58.5	**P = 0.039**	29.3	**P = 0.039**	31.7	**P = 0.026**	36.6	**P = 0.034**	34.1	**P = 0.004**
	Yes	192	88.0		43.8		**33.3**		37.0		**58.9**		**28.1**		**26.0**		**33.3**		**44.8**	
Have you seen someone with leprosy?	No	83	79.5	**P = 0.026**	36.1	P = 0.328	25.3	P = 0.208	41.0	P = 0.904	59.0	P = 0.975	19.3	P = 0.074	19.3	P = 0.140	25.3	P = 0.119	38.6	P = 0.202
	Yes	150	**90.0**		46.0		36.7		38.0		58.7		33.3		31.3		38.7		45.3	
Do you know someone with leprosy?	No	125	81.6	**P = 0.026**	43.2	P = 0.966	33.6	P = 0.894	38.4	P = 0.954	60.8	P = 0.735	24.8	P = 0.432	24.0	P = 0.523	30.4	P = 0.471	43.2	P = 0.797
	Yes	108	**91.7**		41.7		31.5		39.8		56.5		32.4		30.6		38.0		42.6	
Do you have a relative who has/had leprosy?	No	207	86.0	P = 0.730	41.5	P = 0.122	30.4	P = 0.094	40.1	P = 0.170	59.9	P = 0.561	27.1	P = 0.360	27.1	P = 0.817	33.3	P = 0.686	43.0	P = 0.667
	Yes	26	88.5		50.0		50.0		30.8		50.0		38.5		26.9		38.5		42.3	
Think leprosy is due to punishment for sin	No	214	85.5	P = 0.263	44.4	P = 0.085	34.6	P = 0.067	39.7	P = 0.552	**61.7**	**P = 0.005**	30.8	**P = 0.011**	27.6	P = 0.634	35.0	P = 0.336	43.5	P = 0.608
	Yes	19	94.7		21.1		10.5		31.6		26.3		0.0		21.1		21.1		36.8	
Think leprosy is from bad or unclean blood	No	196	84.7	P = 0.109	43.4	P = 0.412	34.7	P = 0.154	38.3	P = 0.509	61.2	P = 0.095	30.1	P = 0.206	26.5	P = 0.588	36.2	P = 0.115	45.4	P = 0.206
	Yes	37	94.6		37.8		21.6		43.2		45.9		18.9		29.7		21.6		29.7	
Think leprosy is a curse	No	174	85.1	P = 0.357	39.1	P = 0.189	31.0	P = 0.317	39.7	P = 0.820	60.9	P = 0.161	27.0	P = 0.731	24.1	P = 0.117	35.1	P = 0.310	45.4	P = 0.333
	Yes	59	89.8		52.5		37.3		37.3		52.5		32.2		35.6		30.5		35.6	
Think leprosy is hereditary	No	198	85.4	P = 0.336	44.4	P = 0.355	34.8	P = 0.223	39.4	P = 0.966	60.6	P = 0.410	30.3	P = 0.277	27.8	P = 0.805	35.4	P = 0.529	46.5	**P = 0.033**
	Yes	35	91.4		31.4		20.0		37.1		48.6		17.1		22.9		25.7		**22.9**	
Think leprosy is caused by marrying from a family that has/had a leprosy patient	No	207	87.4	P = 0.142	43.5	P = 0.656	34.3	P = 0.270	40.6	P = 0.391	60.4	P = 0.352	30.4	P = 0.119	27.5	P = 0.746	35.3	P = 0.409	44.0	P = 0.632
	Yes	26	76.9		34.6		19.2		26.9		46.2		11.5		23.1		23.1		34.6	
Think leprosy is caused by a germ or a microbe	No	169	82.8	**P = 0.014**	42.0	P = 0.311	34.3	P = 0.219	37.3	P = 0.242	55.6	P = 0.147	28.4	P = 0.374	25.4	P = 2.83	30.8	P = 0.123	39.6	P = 0.243
	Yes	64	**95.3**		43.8		28.1		43.8		67.2		28.1		31.2		42.2		51.6	
Think leprosy is caused by living in closed contact with an untreated leprosy patient.	No	209	86.6	P = 0.659	43.5	P = 0.586	33.5	P = 0.573	40.7	P = 0.312	61.7	**P = 0.027**	30.1	P = 0.165	27.8	P = 0.620	36.8	**P = 0.018**	45.0	P = 0.168
	Yes	24	83.3		33.3		25.0		25.0		**33.3**		12.5		20.8		**8.3**		25.0	
Think leprosy is due to poor personal hygiene	No	166	88.0	P = 0.239	43.3	P = 0.698	37.3	**P = 0.042**	42.8	P = 0.124	59.6	P = 0.804	31.9	P = 0.131	27.7	P = 0.837	39.2	**P = 0.022**	49.4	**P = 0.007**
	Yes	67	82.1		40.3		**20.9**		29.9		56.7		19.4		25.4		**20.9**		**26.9**	
Think leprosy occurs spontaneously	No	213	86.4	P = 0.863	43.7	P = 0.321	33.8	P = 0.322	39.9	P = 0.465	59.2	P = 0.697	29.1	P = 0.509	27.7	P = 0.568	34.3	P = 0.707	43.2	P = 0.695
	Yes	20	85.0		30.0		20.0		30.0		55.0		20.0		20.0		30.0		40.0	
Think leprosy is curable	No	58	69.0	**P < 0.001**	34.5	**P = 0.002**	31.0	**P = 0.016**	24.1	**P < 0.001**	39.7	**P < 0.001**	29.3	**P = 0.014**	25.9	**P = 0.016**	24.1	**P = 0.005**	34.5	**P = 0.002**
	Yes	175	**92.0**		**45.1**		**33.1**		**44.0**		**65.1**		**28.0**		**27.4**		**37.1**		**45.7**	

Figures under the question columns represent percentages of participants with a "Yes" response to the question

### Independent predictors of attitudes towards PWL

In a binary logistic regression inputting community perceptions and knowledge that influenced attitudes with respect to leprosy, seven independent predictors were identified (Table 6). The positive attitude of advising a relative or friend to seek treatment from a health facility was enhanced by two predictors: the understanding that leprosy is caused by a germ, and that it is curable.

**Table 6:** Independent predictors of attitudes towards PWL.

Attitudes	Independent Predictors		95% CI		
		OR	Lower	Upper	P-value
**Would advise relative to seek treatment at a health facility or from a health worker**	- Think leprosy is caused by a germ	3.86	1.11	13.48	0.034
	- Think leprosy is curable	4.93	2.24	10.87	<0.001
**Would not shake hands with someone with leprosy**	- Think leprosy is a curse	2.10	1.12	3.95	0.021
	- Think leprosy is a punishment for sin	0.25	0.08	0.81	0.021
**Would not eat from the same plate with someone who has leprosy**	- Has seen a leprosy patient	2.09	1.12	3.87	0.02
	- Think leprosy is due to poor personal hygiene	0.37	0.19	0.74	0.005
**Would feel ashamed if he/she had leprosy**	- Think leprosy is curable	2.64	1.34	5.21	0.005
	- Think leprosy is due to poor personal hygiene	0.52	0.28	0.97	0.039
**Would not reveal status to anyone if he/she had leprosy**	- Think leprosy is a punishment for sin	0.22	0.08	0.64	0.005
**Would not allow child to marry from a family with a history of leprosy**	- Has seen a leprosy patient	1.90	1.00	3.60	0.049
**Would not allow child to play with another child who has leprosy**	- Has seen a leprosy patient	2.63	1.34	5.13	0.005
	- Think leprosy is due to poor personal hygiene	0.46	0.22	0.96	0.038
**Think that people with leprosy should not be allowed to participate in activities like anyone else**	- Has seen a leprosy patient	2.42	1.28	4.60	0.007
	- Think leprosy is caused by a germ	2.78	1.38	5.61	0.004
	- Think leprosy is due to poor personal hygiene	0.32	0.14	0.71	0.005
**Think that people with leprosy should not be given employment like anyone else**	- Has seen a leprosy patient	1.85	1.02	3.37	0.044
	- Think leprosy is caused by a germ	3.38	1.65	6.93	0.001
	- Think leprosy is hereditary	0.31	0.12	0.79	0.014
	- Think leprosy is due to poor personal hygiene	0.27	0.13	0.56	<0.001

The eight negative attitudes studied (Table 6) were driven by three independent predictors, namely: having seen a leprosy patient, the belief that leprosy is a curse, and the knowledge that it is caused by a germ. However, the effect of these negative attitudes was dampened by three predictors namely: the knowledge that leprosy is due to poor personal hygiene or the beliefs that it is a punishment or that it is hereditary, which were found to be protective.

## Discussion

Although the WHO enhanced global strategy for further reducing the burden of leprosy for the period 2011–2015 [20] has been implemented in Cameroon, over 300 new cases of leprosy continue to be reported in the country each year [21]. A new WHO global leprosy strategy 2016–2020 has been launched and has as main focus: the reduction of leprosy transmission and of leprosy related disabilities, stigma and discrimination [29]. The implementation of this strategy could face the hurdle of lack of community knowledge, and erroneous perceptions about leprosy [15]. The success of any intervention to improve upon the outcomes of leprosy control would depend on a good understanding of these community knowledge and perceptions [15].

In the current study, 82.4% of respondents had heard about leprosy. Though relatively high, this figure is less than the 100% reported in an Ethiopian study [30]. The sources of community information on leprosy in our study were varied (Fig 1). The most important sources of information were from community volunteers, friends and the media and only to a lesser extent from health personnel and schools. In Cameroon, community relay agents (volunteers) are important stake-holders in community health programmes like vaccination, community distribution of ivermectine against onchocerciasis and distribution of treated bed nets in the fight against malaria, and Buruli ulcer control [31; 32]. From our findings, an intervention to address community awareness on leprosy through the community relay agents, and local community radios could be the most effective approach.

Only 19.7% of our participants knew the cause of leprosy. This is comparable to the 19.26% reported in Ethiopia [30], but better than the 0% reported in a community in Pakistan [33]. The majority of our participants wrongly cited as causes of leprosy: curse, bad blood, heredity, punishment for sins, and eating some types of food (Table 3). Similar misconceptions have been reported in the northwest of Cameroon [13]. In Ethiopia it is believed that leprosy is linked to curse/punishment by god, heredity, bad blood, and immoral conduct [30], while in eastern Sudan it has been linked mainly to some food types [34]. These misconceptions are clearly grounded in the customs and beliefs of the communities concerned, and are common to cultures in Africa, Asia and South America [15].

Seventy-five percent of our participants knew that leprosy is curable. This is higher than the 67.9% reported in Mezam division in the northwest of Cameroon [35], 60% in Mangalore-India [36] and 18.3% in Pakistan [33], but less than the 92.5% reported in Ethiopia [30]. In our sample, business men (P = 0.043) and those with a high school or university education (P = 0.047), were most likely to know that leprosy is curable. Furthermore, 86.3% would refer a relative or friend with leprosy to a health facility for treatment. A comparable finding was reported in India [36]. This practice was strongly influenced by the knowledge that leprosy is curable (P<0.001), the understanding that leprosy is caused by a germ (P = 0.014), or knowing someone with leprosy (P = 0.026). A considerable proportion (43% to 71%) of our respondents acknowledged that PWL face various and varied challenges in the society. At the individual patient level, the challenges range from difficulties in getting employment, getting admission in schools, interacting with other people, to getting married. The challenges went beyond the individual patient to affect the patient’s family like bringing shame to the family, and problems in marriage. The challenges faced by PWL are certainly a reflection of the society’s attitudes towards them.

Attitudes were generally negative in our sample (Tables 4 and 5). The negative attitudes were not influenced by demographic variables in our study, but were strongly influenced by lack of knowledge about leprosy and socio-cultural perceptions of the diseases (Table 5). Similarly, negative attitudes towards PWL have been reported in Ethiopia [30], and Secunderabad, India [37].

One positive and eight negative attitudes were found in our study. The lone positive attitude of advising a relative or friend with leprosy to seek medical treatment was independently driven by the knowledge that leprosy is caused by a germ, and that it is curable. This finding has important public health implications. The ultimate goal of any leprosy control programme is to break the transmission chain in endemic communities. This can only happen if all detected leprosy patients are treated adequately with multi-drug-therapy against leprosy. Increasing community knowledge on these two aspects regarding leprosy is therefore paramount.

The independent predictors of negative attitudes were: having seen a leprosy patient, the knowledge that leprosy is caused by a germ and the belief by some that it is a curse. In the Oroko language, the name for leprosy is “diangi” signifying a disease that cuts off fingers, toes and destroys the face. With this kind of perception about leprosy, community members develop fear of being infected and becoming a leper, if they associated with PWL. The common tendency is therefore to avoid PWL in all circumstances.

The knowledge that leprosy is due to poor personal hygiene or the beliefs that it is a punishment for sins or is hereditary, were found to be independently protective against some negative attitudes in this study. Some community members tend to pity PWL and would not support some of the negative attitudes like refusing to shake hands with PWL; not allowing their child to play with PWL; or their relative to marry from a family with history of leprosy, on the basis that leprosy is due to poor personal hygiene. In rural communities of Cameroon, environmental and personal hygiene are generally poor, with very poor housing conditions and limited access to potable water [38] which is not limited only to PWL. In our study, some community members also did not see why PWL should not be employed, on the basis of the belief that leprosy is hereditary.

We conclude that familiarity with leprosy was very high, with the major sources of information being from community volunteers and the media. However, knowledge on the cause of leprosy was very low, with a considerable proportion having erroneous perceptions about its cause. Quite a high proportion of our participants understood that leprosy is curable and would refer their relatives or friends with leprosy for medical treatment.

Attitudes toward PWL were very negative in our sample. These negative attitudes were independently driven by the perception that leprosy is a curse, the knowledge that leprosy is caused by a germ, and having seen a leprosy patient. The negative attitudes were however dampened by the beliefs that leprosy is a punishment, is hereditary or is due to poor personal hygiene.

We recommend that, a leprosy awareness intervention, through the channel of community volunteers and the media, with information on the correct cause of leprosy, its curable nature, and messages discouraging the erroneous perceptions regarding it, could improve upon the community knowledge of leprosy, as well as attitudes towards PWL. This could ultimately lead to the reduction of leprosy burden in this community.

## Supporting information

S1 Data Compressed shapefiles of Cameroon health district, used for drawing up the map of Ekondotiti and Mbonge health districts, highlighting the villages visited and number of participants in each village (Fig 1). (RAR)

S1 Text STROBE checklist for this cross-sectional study. (DOC)
