# Supplementary material for: Community knowledge, perceptions and attitudes regarding leprosy in rural Cameroon: The case of Ekondotiti and Mbonge health districts in the South-west Region
Source: PLoS Negl Trop Dis. 2018 Feb 12;12(2):e0006233. doi: 10.1371/journal.pntd.0006233 (PMC5825164; doi:10.1371/journal.pntd.0006233)
Supplement: S1 Text — (DOC) [file pntd.0006233.s002.doc]

STROBE Statement—Checklist of items that should be included in reports of ***cross-sectional studies***

|  | Item No | Paragraph (Line number) |
| --- | --- | --- |
| **Title and abstract** | 1 | Paragraph 1-4 (line 1-24) |
| Introduction | |  |
| Background/rationale | 2 | Paragraph 10-14 (line 41-74) |
| Objectives | 3 | Paragraph 15 (line 75-78) |
| Methods | |  |
| Study design | 4 | Paragraph 16 (line 79-83) |
| Setting | 5 | Paragraph 17-19 (line 84-104) |
| Participants | 6 | Paragraph 20-21 (line 105-112) |
| Variables | 7 | Paragraph 23-26) (line 124-133) |
| Data sources/ measurement | 8* | Paragraph 22 (Line 113-123) |
| Bias | 9 |  |
| Study size | 10 | Paragraph 27 (line 134-136) |
| Quantitative variables | 11 | See item No 7 |
| Statistical methods | 12 | Paragraph 28-29 (line 141-148) |
| Results | |  |
| Participants | 13* | Paragraph 30 (line 149-154) |
| Descriptive data | 14* | Paragraph 30 (line 149-154) |
| Outcome data | 15* | Paragraph 31-37 (line 155-220) |
| Main results | 16 | Paragraph 31-37 (line 155-220) |
| Other analyses | 17 | Paragraph 38-39 (line 221-230) |
| Discussion | |  |
| Key results | 18 | Paragraph 140-147 (line231 -302) |
| Limitations | 19 |  |
| Interpretation | 20 | Paragraph 140-147 (line231 -302) |
| Generalisability | 21 | Paragraph 148-150 (Line 303-317) |
| Other information | |  |
| Funding | 22 | Paragraph 151 (line 318-320) |

*Give information separately for exposed and unexposed groups.

**Note:** An Explanation and Elaboration article discusses each checklist item and gives methodological background and published examples of transparent reporting. The STROBE checklist is best used in conjunction with this article (freely available on the Web sites of PLoS Medicine at http://www.plosmedicine.org/, Annals of Internal Medicine at http://www.annals.org/, and Epidemiology at http://www.epidem.com/). Information on the STROBE Initiative is available at www.strobe-statement.org.
